# Non-Volatile Metabolites from *Trichoderma* spp.

**DOI:** 10.3390/metabo9030058

**Published:** 2019-03-22

**Authors:** Meng-Fei Li, Guo-Hong Li, Ke-Qin Zhang

**Affiliations:** State Key Laboratory for Conservation and Utilization of Bio-Resources in Yunnan, and Key Laboratory for Microbial Resources of the Ministry of Education, Yunnan University, Kunming 650091, China; limengfei131420@163.com

**Keywords:** bioactivity, metabolites, *Trichoderma*

## Abstract

The genus *Trichoderma* is comprised of many common fungi species that are distributed worldwide across many ecosystems. *Trichoderma* species are well-known producers of secondary metabolites with a variety of biological activities. Their potential use as biocontrol agents has been known for many years. Several reviews about metabolites from *Trichoderma* have been published. These reviews are based on their structural type, biological activity, or fungal origin. In this review, we summarize the secondary metabolites per *Trichoderma* species and elaborate on approximately 390 non-volatile compounds from 20 known species and various unidentified species.

## 1. Introduction

*Trichoderma* is a genus of fungi of the family Hypocreaceae. It is distributed in soils worldwide across various habitats [1]. *Trichoderma* is a valuable resource for structurally novel natural products with diverse bioactivities [2]. Among well-studied fungi, *Trichoderma* species are known for their ability to produce bioactive secondary metabolites, including polyketides, alkaloids, terpenoids, and peptaibols [3]. Many species have been extensively investigated due to their application as biological control agents [4]. In this article, we reviewed the origin, structure, and bioactivity of non-volatile secondary metabolites from *Trichoderma* spp. and grouped them per species.

## 2. Results

### 2.1. Metabolites from Trichoderma arundinaceum

A series of peptaibols were isolated from the scaled-up fermentation of *T. arundinaceum* MSX70741: three new compounds [prealamethicin F50 (**1**); Glu(OMe)^18^-alamethicin F50 (**2**); and trichobrevin BIII-D (**3**)], and four known compounds [alamethicin F50 (**4**); alamethicin II (**5**); atroviridin J (**6**); and trichobranchin D-I (**7**)]. The cytotoxic activity of compounds **2**, **3**, **4,** and **6** were evaluated against a panel of cancer cell lines: HCT 116, DLD-1, HT-29, SW948, Hep-G2, Huh-7, and HeLa. Compound **2** was the most active compound with IC_50_ values ranging from 2.5 through 6.5 mM. Compound **3** exhibited moderate activity against HCT 116 and HT-29, with IC_50_ values of 6.8 and 6.7 mM, respectively [3].

### 2.2. Metabolites from Trichoderma asperellum

Nine compounds were isolated from the fungus *T. asperellum*: trichodermaerin (**8**) [5]; 6-amyl alpha-pyrone (**9**) [6]; aspereline G (**10**); aspereline H (**11**); aspereline A (**12**); aspereline C (**13**); aspereline D (**14**); aspereline E (**15**); and aspereline F (**16**) [7]. Among them, compounds **10** and **11** were two new peptaibols.

Eight new compounds were isolated from the marine-derived fungus *T. asperellum* cf44-2: bisabolane sesquiterpene bisabolan-1,10,11-triol (**17**); norbisabolane sesquiterpene 12-nor-11-acetoxybisabolen-3,6,7-triol (**18**); two naturally occurring monoterpenes [(7*S*)-1-hydroxy-3-*p*-menthen-9-oic acid (**19**) and (7*R*)-1-hydroxy-3-*p*-menthen-9-oic acid (**20**)]; trichodenone dechlorotrichodenone C (**21**); chlorine-containing trichodenone 3-hydroxytrichodenone C (**22**); diketopiperazine methylcordysinin A (**23**); and oxazole derivative 4-oxazolepropanoic acid (**24**). Compounds **17**, **18**, **21** and **22** were evaluated for the inhibition of four marine phytoplankton species (*Chattonella marina*, *Heterosigma akashiwo*, *Karlodinium veneficum*, and *Prorocentrum donghaiense*) and four marine-derived pathogenic bacteria (*Vibrio parahaemolyticus*, *V. anguillarum*, *V. harveyi*, and *V. splendidus*). All exhibited growth inhibition of the four phytoplankton species, and compound **18,** with IC_50_ values ranging from 4.2 to 8.5 µg/mL, was more active than the others. Additionally, compounds **17**, **18**, **21** and **22** showed weak antibacterial activities against the four *Vibrio* species, with inhibitory zone diameters of 6.2–8.5 mm at 20 µg/disk. Among them, compound **18** had the highest antibacterial activity [8].

From the cultures of *T. asperellum* dl-34, eighteen compounds were identified: a new diterpenoid, wickerol A (**25**); a known diterpenoid, harziandione (**26**); ten known steroids [ergosterol endoperoxide (**27**); 5α,8α-epidioxyergosta-6,9(11),22-trien-3β-ol (**28**); 3β,5α,6β-trihydroxyergosta-7,22-diene (**29**); 3β,5α-dihydroxy-6β-methoxyergosta-7,22-diene (**30**); 3β,5α,9α-trihydroxyergosta-7,22-dien-6-one (**31**); (22*E*,24*R*)-ergosta-4,6,8,(14),22-tetraen-3-one (**32**); (22*E*,24*R*)-5α,6α-epoxyergosta-8,22-diene-3β,7α-diol (**33**); ergosta-7,22-dien-3β-ol (**34**); (22*E*,24*R*)-ergosta-5,7,22-trien-3β-ol (**35**); and β-sitosterol (**36**)]; two diketopiperazines, [(L)-Pro-(L)-Leu (**37**) and (L)-4-OH-Pro-(L)-Leu] (**38**); one nucleotide, adenine nucleoside (**39**); and three polyketides, [cis-4-hydroxy-6-deoxyscytalone (**40**); 2,4-dihydroxy-3,6-dimethylbenzaldehyde (**41**); and dihydrocitrinone (**42**)]. Most of these compounds were screened for biological activities, only compounds **25** and **26** were toxic to *Artemia salina*, with LC_50_ values of 12.0 and 38.2 μg/mL, respectively [9].

### 2.3. Metabolites from Trichoderma atroviride

Eleven compounds were obtained from the marine-derived fungus *T. atroviride*: three novel compounds [3-amino-5-hydroxy-5-vinyl-2-cyclopenten-1-one dimer atrichodermone A (**43**); cyclopentenone derivative atrichodermone B (**44**), and sesquiterpene atrichodermone C (**45**) [10]] and eight known compounds [atrichodermone D (**46**); trichodermone A (**47**); (5R)5-hydroxy-3-[(methoxycarbonyl)-amino]-5-vinyl-2-cyclopenten-1-one (**48**); 4*H*-1,3-dioxin-4-one-2,3,6-trimethyl (**49**); 1,3-dione-5,5-dimethylcyclohexane (**50**); 2-enone-3hydroxy-5,5-dimethylcylohex (**51**) [11]; 6-pentyl-pyran-2-one (**52**); and 6-pent-1-enyl-pyrane-2-one (**53**) [1]]. Among these, compounds **43**–**45** were evaluated for their cytotoxicity against HL60 and U937 cell lines, as well as anti-inflammatory effect against the production of the pro-inflammatory cytokines TNF-α and IL-1β; but none showed notable cytotoxicity or anti-inflammatory activity. Compound **49** significantly inhibited the growth of *Helicobacter pylori* and Shigella toxin-producin*g Escherichia coli*, and it also induced cell death and cytotoxicity.

Five new compounds were isolated from the marine-derived fungus *T. atroviride* G20-12: 2-hydroxybutan-3-yl5′-(2″-hydroxy-N-(2‴-oxobutan-3‴-yl)propanamido)butanoate (**54**); 3-hydroxy-5-(4-hydroxybenzyl)dihydrofuran-2(3*H*)-one (**55**) [12]; 4′-(4,5-dimethyl-1,3-dioxolan-2-yl)methyl-phenol (**56**); (3′-hydroxybutan-2′-yl)5-oxopyrrolidine-2-carboxylate (**57**) and atroviridetide (**58**) [13].

Eight compounds were isolated from the solid culture of endophytic fungus *T. atroviride* S361: a pair of novel N-furanone amide enantiomers [(-)-trichodermadione A (**59a**) and (+)-trichodermadione A (**59b**)]; a new cyclohexenone sesquiterpenoid, trichodermadione B (**60**); and six known compounds [4-(2-formyl-5-(methoxymethyl)-1*H*-pyrrol-1-yl)butanoic acid (**61**); 5-methoxymethyl-1*H*-pyrrole-2-carbaalde-hyde (**62**); 3-(1-carbaalde)-6-methyl-2*H*-pyran-2,4(3*H*)-dione (**63**); lignoren (**64**); ascotrichic acid (**65**); and catenioblin C (**66**). Compounds **59** and **60** were also evaluated for their cytotoxicity against DU145 and PC3 cell lines, as well as inhibitory effects against the production of NO in lipopolysaccharide (LPS)-stimulated RAW264.7 cells. However, none of them showed notable cytotoxicity or anti-inflammatory activity [14].

The compound 6-pentyl-α-pyrone (**67**), which was isolated from *T. atroviride* UST1 and UST2, it was involved in *Trichoderma*-pathogen interactions on grapevine pruning wounds [15].

### 2.4. Metabolites from Trichoderma aureoviride

A new compound, koninginin G (**68**), and a known compound, Koninginin G triacetate (**69**), were obtained from a strain of *T. aureoviride*. Compound **68** significantly inhibited the growth of etiolated wheat coleoptiles by 56% at 10^−3^ M concentration [16].

### 2.5. Metabolites from Trichoderma brevicompactum

The bioactive compound trichodermin (**70**) was isolated from the endophytic fungus *T. brevicompactum.* It displayed significant inhibitory activity on *Rhizoctonia solani* and *Botrytis cinerea*, with an EC_50_ of 0.25 μg/mL and 2.02 μg/mL, respectively. However, a relatively poor inhibitory effect was shown against *Colletotrichum lindemuthianum* (EC_50_ = 25.60 μg/mL) [17].

### 2.6. Metabolites from Trichoderma citrinoviride

Fifteen compounds were isolated from the *T. citrinoviride:* four new compounds [(*R*)-vertinolide (**71**) [1]; trichoderiol C (**72**); citrinoviric acid (**73**); and penicillenol D (**74**)] and twelve known compounds [lignoren (**64**); trichotetronine (**75**); bisvertinol (**76**); spirosorbicillinol A (**77**); spirosorbicillinol B (**78**); spirosorbicillinol C (**79**); trichoderiol A (**80**); penicillenol B_1_ (**81**); penicillenol B_2_ (**82**); cyclo-(Leu-Pro) (**83**); cyclo-(Ile-Pro) (**84**); and cyclo-(Phe-Pro) (**85**)] [18]. Among them, compounds **73** and **74** showed moderate cytotoxic effects against the A-375 cell line, with IC_50_ values of 85.7 and 32.6 μM, respectively.

From *T. citrinoviride* cf-27, twenty-two metabolites were obtained: a new diterpene, trichocitrin (**86**), and twenty-one known compounds [ergosterol endoperoxide (**27**); (22*E*,24*R*)-ergosta-4,6,8,(14),22-tetraen-3-one (**32**); (22*E*,24*R*)-ergosta-5,7,22-trien-3β-ol (**35**); 24-methylenecycloartanol (**87**); cycloeucalenol (**88**); citrostadienol (**89**); euphorbol (**90**); 24-methylene-lanost-8-en-3β-ol (**91**); cyclonerodiol (**92**); (22*E*,24*R*)-7β,8β-epoxy-3β,5α,9α-trihydroxyergosta-22-en-6-one (**93**); nafuredin (**94**); harzianolide (**95**); 5-hydroxy-2,3-dimethyl-7-methoxychromone (**96**); 5-hydroxy-3-hydroxymethyl-2-methyl-7-methoxychromone (**97**); methyl 8-hydroxy-6-methyl-9-oxo-9*H*-xanthene-1-carboxylate (**98**); methyl 2,8-dihydroxy-6-methyl-9-oxo-9*H*-xanthene-1-carboxylate (**99**); stachyline B (**100**); trans-3,4-dihydro-2,4,8-trihydroxynaphthalen-1(2*H*)-one (**101**); pyrazole-3-carboxylic acid (**102**); pyrrole-2-carboxylic acid (**103**); and dibutyl phthalate (**104**)]. Most of the isolated compounds were screened for biological activities, and the results showed that compounds **86** and **94** exhibited 54.1% and 36.7% inhibition, respectively, of *P. donghaiense* at 100 μg/mL [9].

### 2.7. Metabolites from Trichoderma cremeum

A new 10-member lactone, cremenolide (**105**), was isolated from *T. cremeum*. In vitro tests showed that cremenolide inhibited the radial mycelium growth of *Fusarium oxysporum*, *B. cinerea*, and *R. solani*, and it significantly promoted tomato seedling growth [19].

### 2.8. Metabolites from Trichoderma gamsii

Two new cytochalasans, trichoderones A (**106**) and B (**107**), and three known analogues, aspochalasins D (**108**), J (**109**), and I (**110**), were isolated from the endophytic fungus *T. gamsii*. Compound **106** possesses an unprecedented 7/6/6/5/5 pentacyclic system, whereas compound **107** contains the rare 6/5/6/6/5 pentacyclic skeleton with a 12-oxatricyclo [6.3.1.0^2,7^] moiety. Compounds **108** and **109** displayed cytotoxic activity against the HeLa cell line [20].

### 2.9. Metabolites from Trichoderma harzianum

Fourteen compounds were identified from the cultures of *T. harzianum* R5: ergosterol endoperoxide (**27**); 5α,8α-epidioxyergosta-6,9(11),22-trien-3β-ol (**28**); 3β,5α,6β-trihydroxyergosta-7,22-diene (**29**); adenine nucleoside (**39**); trichoharzianin (**111**); 3β-hydroxyergosta-8,24(28)-dien-7-one (**112**); (22*E*,24*R*)-24-methylcholesta-5,22-dien-3β-ol (**113**); 5,7-dihydroxy-2,3-dimethylchromone (**114**); (22*E*,24*R*)-3β,5α-dihydroxy-ergosta-7,22-dien-6-one (**115**); 5-hydroxy-2-hydroxymethyl-3-methyl-7-methoxychromone (**116**); indole-3-carboxaldehyde (**117**); 3-indol acetic acid (**118**); 2,4-dimethylbenzene-1,3,5-triol (**119**); and 5′-*o*-acetyluracil nucleoside (**120**). Compound **111** was a new terpenoid that showed significant lethal activity against *A. salina*, and the LC_50_ value was 68.6 μg/mL [9].

Five terpenoids [cyclonerodiol (**92**); wickerol B (**121**); 15-hydroxyacorenone ((1*S*,4*S*,5*S*)-8-hydroxymethyl-1-isopropyl-4-methyl spiro[4.5]dec-8-en-7-one) (**122**); epicycloneodiol oxide (**123**); and cycloneodiol oxide (**124**)], one lactone [5,6-dihydro-4-methyl-2*H*-pyran-2-one (**125**)], and one steroid [demethylincisterol A3 (**126**)] from *T. harzianum* R5-1 were studied. Three bacterial strains (*V. splendidus*, *V. arveyi*, and *V. anguillarum*) were tested for resistance to these compounds. Compounds **92**, **121**, **122**, **123,** and **124** showed an inhibitory effect on *V. anguillarum* [21].

Six compounds were isolated from *T. harzianum* T-4: β-sitosterol (**36**); palmitic acid (**127**); 1,8-dihydroxy-3-methylanthraquinone (**128**); 6-pentyl-2*H*-pyran-2-one (**129**); 2(5*H*)-furanone (**130**); and stigmasterol (**131**). While seven were isolated from *T. harzianum* strain T-5: palmitic acid (**127**); 6-pentyl-2*H*-pyran-2-one (**129**); 1-hydroxy-3-methylanthraquinone (**132**); δ-decanolactone (**133**); ergosterol (**134**); harzianopyridone (**135**); and 6-methyl-1,3,8-trihydroxyanthraquinone (**136**). These compounds were screened for antifungal activity; compound **135** was the most active, with an EC_50_ of 35.9–50.2 mg/mL [22].

Harzianolide (**95**); 1,8-dihydroxy-3-methylanthraquinone (**128**); 1-hydroxy-3-methylanthraquinone (**132**); harzianopyridone (**135**); T22azaphilone (**137**); and T39butenolide (**138**) were obtained from the broth of *T. harzianum* T22 and *T. harzianum* T39. In antifungal assays, compounds **135** and **137** inhibited the growth of *Leptosphaeria maculans, Phytophthora cinnamomi*, and *B. cinerea* even at low doses (1–10 μg per plug), while high concentrations of compounds **95** and **138** were needed (>100 μg per plug) for inhibition [23].

Six compounds were isolated from *T. harzianum* dl-36: 5α,8α-epidioxyergosta-6,9(11),22-trien-3β-ol (**28**); 3β,5α,9α-trihydroxyergosta-7,22-dien-6-one (**31**); (22*E*,24*R*)-ergosta-5,7,22-trien-3β-ol (**35**); harzianolide (**95**); (22*E*,24*R*)-5α,8β-epidioxyergosta-6,22-dien-3-β-ol (**139**); and ergosta-7,22-dien-3β,5α,6β-triol (**140**) [24].

Thirty-two compounds were obtained from *T. harzianum*: 6-pentyl-pyran-2-one (**52**); trichodermin (**70**) [25,26]; cyclonerodiol (**92**); harzianic acid (**141**) [27]; 15-hydroxyacorenone (**142**) [28]; 2460A (**143**) [29]; trichokindins I–VII (**144**–**150**) [30]; trichorozins I–IV (**151**–**154**) [31]; octaketide keto diol (**155**) [32]; oxidized analog (**156**) [1]; 2-phenylethanol (**157**); tyrosol (**158**); 6-n-pentyl-•-pyrone (1**59**) [33]; cyclo-(R-Pro-Gly) (**160**); cyclo-(R-Pro-R-Ala) (**161**); cyclo-(S-Pro-R-Va1) (**162**); cyclo-(4-methyl-R-Pro-S-Nva) (**163**); cyclo-(R-Pro-R-Leu) (**164**); cyclo-(R-Pro-R-Phe) (**165**); cyclo-(4-hydroxyl-S-Pro-S-Leu) (**166**); uraci (**167**); p-hydroxylphenylethanol (**168**); and m-hydroxylphenylacitic acid (**169**) [34]. Compound **70** exhibited antifungal activity against the mycelial growth of *F. oxysporum*, *C. lindemuthianum*, *C. gloeosporioides*, *Thanatephorus cucumeris*, *R. solani*, *B. cinerea*, and *Cochliobolus miyabeanus.* It also prevented the spore germination of pathogenic fungi *T. cucumeris* and *R. solani*. Compound **141** showed antibiotic activity against *Pythium irregulare*, *Sclerotinia sclerotiorum*, and *R. solani*; and a plant-growth-enhancing effect was observed at low concentrations. The anti-tumor activities of the new compound **143** was demonstrated on CM126 and HT-29 cell lines, with an IC_50_ of 2.17 × 10^−5^ mol/L and 1.8 × 10^−5^ mol/L respectively; and the compound somewhat affected the HT-29 cell cycle at S phase. Seven new peptaibols, compounds **144**–**150**, induced Ca^2+^-dependent catecholamine secretion from bovine adrenal medullary cells. Compound **159** showed antifungal and antibacterial activity and completely inhibited the growth of fungus *Armillaria mellea* at a concentration of 200 ppm. Compounds **160**–**169** were isolated from *T. harzianum* for the first time.

In addition, compound 6-pentyl-α-pyrone (**67**) was also found from *T. harzianum* T77 and SQR-T037. It is used for the control of grapevine trunk diseases [15], and it effectively controlled *F. oxysporum* and may control *Fusarium* wilt in cucumber, in continuously cropped soil [35].

### 2.10. Metabolites from Trichoderma koningii

An unstable antifungal compound, 3-dimethylamino-5-hydroxy-5-vinyl-2-cyclopenten-l-one (1**70**), which was a new cyclopentenone derivative, was obtained from the marine-derived fungus *T. koningii* [36]. From another marine fungus *T. koningii*, five new polyketide derivatives, 7-*O*-methylkoninginin D (**171**) and trichodermaketones A–D (**172**–**175**), together with four known compounds, koninginin A (**176**); koninginin D (**177**); koninginin E (**178**); and koninginin F (**179**), were identified [36]. Compound **172** showed synergistic antifungal activity against *Candida albicans* with 0.05 µg/mL ketoconazole [37].

Four compounds were isolated from *T. koningii* T-8: palmitic acid (**127**); δ-decanolactone (**133**); 6-pentyl-α-pyranone (**180**); and 6-(4-oxopentyl)-2*H*-pyran-2-one (**181**). Two compounds, stigmasterol (**131**) and 6-pentyl-α-pyranone (**180**), were obtained from *T. koningii* T-11. These compounds were evaluated for antifungal activity against soilborne pathogenic fungi *R. solani*, *Sclerotium rolfsii*, *Macrophomina phaseolina*, and *F. oxysporum*. Compounds **180** and **181** exhibited excellent antifungal activity against *S. rolfsii* [38].

Fourteen metabolites were derived from *T. koningii*: which included a new sesquiterpene alcohol, tricho-acorenol (**182**) [39], and thirteen other compounds: cyclonerodiol (**92**); uracil (**167**); methyl benzoate (**183**); cyclo-(L-Pro-L-Leu) (**184**); 4-hydroxyphenethylalcohol (**185)**; ceramide (**186**); and trichokonins-V, VI, II, III, Ia, Ib, and IX (**187**–**193**) [40,41].

### 2.11. Metabolites from Trichoderma koningiopsis

Four koninginin compounds were characterized from *T. koningiopsis* [1]: trikoningin KAV (**194**); 11-residue lipopeptaibols (**195**); trikoningin KB I (**196**); and trikoningin KB II (**197**).

Five polyketides were isolated from *T. koningiopsis* YIM PH30002. Their structures were elucidated as konginginin A (**198**); konginginin B (**199)**; konginginin D (**200**); konginginin F (**201**); and konginginin M (**202**) [42]. Among them, compounds **198**–**201** showed siderophoric activity. Compound **199** presented higher activity with a maximum tolerable concentration of 300 μg/mL, in the iron (Fe III) acquisition tests. Compounds **198**–**202** exhibited weak antimicrobial activity against *Acinetobacter baumanii*, *Staphylococcus aureus*, *F. oxysporum*, *F. solani* and *Alternaria panax.*

Twenty-four compounds were identified from *T. koningiopsis* Y10-2 [43]: wickerol A (**25**); harziandione (**26**); cyclonerodiol (**92**); wickerol B (**121**); epicycloneodiol oxide **(123**); cycloneodiol oxide (**124**); koninginin A (**176**); koninginin D (**177**); 3-acetyl-6-methyl-2*H*-pyran-2,4(3*H*)-dione (**203**); lutidonecarboxylic acid (**204**); cyclonertriol (**205**); 2-hydroxydiplopterol (**206**); verrucosidin (**207**); neoechinulin A (**208**); isoechinulin A (**209**); echinuline (**210**); cyclo-trans-4-OH-(D)-Pro-(D)-Phe (**211**); fructigenine A (**212**); 3-*o*-methylviridicatin (**213**); cyclopenol (**214**); olemolide (**215**); ethyl 4-hydroxyphenylacetate (**216**); 4-hydroxyphenylethanol (**217**); and m-methoxyphenol (**218**). A preliminary evaluation on antibacterial and antimicroalgal activities, as well as brine shrimp lethality of some compounds were carried out. The results showed that compound **214** displayed excellent activity against *Pseudoalteromonas citrea*, *V. parahaemolyticus*, *V. splendidus*, *V. anguillarum*, and *V. harveyi*, with IC_50_ values ranging from 8 to 32 μg/mL. Compounds **209**, **210,** and **212** showed potent inhibitory activity against *C. marina*, *P. donghaiense, H. akashiwo*, and *K. veneficum*, with IC_50_ values ranging from 0.040 to 12 μg/mL.

### 2.12. Metabolites from Trichoderma lignorum

Lignoren (**64**), a new sesquiterpenoid, was first isolated from *T. lignorum* HKI 0257. It showed moderate antimicrobial activity against *Bacillus subtilis* ATCC 6633, *Mycobacterium smegmatis* SG 987, and *Pseudomonas aeruginosa* K 599/WT [44].

### 2.13. Metabolites from Trichoderma longibrachiatum

Eight known compounds were identified from the marine-derived endophytic *T. longibrachiatum*: β-sitosterol (**36**); ergosterol (**134**) [33]; sorbicillin (**219**); ergosterol peroxide (**220**); cerevisterol (**221**); 2-anhydromevalonic acid (**222**); squalene (**223**) [45]; and ergokonin A (**224**) [46]. Biological activity indicated that compound **219** exhibited moderate activity against *Bacillus brevis*, *B. subtilis*, *Sarcina lutea*, and *Enterobacter dissolvens*. Compound **224** exhibited activity against *Candida* and *Aspergillus* species but was inactive against *Cryptococcus* species; and it induced alterations in the hyphal morphology of *Aspergillus fumigatus*.

Two new tetronic acid derivatives were isolated from *T. longibrachiatum* Rifai aggr, 5-hydroxyvertinolide (**225**) and bislongiquinolide (**226**), which were antagonistic to the fungus *Mvcena citricolor* [47].

A new sesquiterpene, 10,11-dihydrocyclonerotriol (**227**), together with two known compounds, catenioblin C (**66**) and sohirnone A (**228**), were identified from the endophytic fungus *T. longibrachiatum* YM311505. Compounds **66**, **227** and **228** exhibited antifungal activities against *Pyricularia oryzae* and *C. albicans* [48].

Two compounds, trichokonins A (**229**) and B (**230**), were obtained from *T. longibrachiatum* SMF2. Compound **229** exhibited a variety of biological activities: antimicrobial, antiviral, anti-tumor, and inducing plant resistance [49].

### 2.14. Metabolites from Trichoderma polysporum

A new minor metabolite valinotricin (**231**) was reported from *T. polysporum*, along with cyclonerodiol oxide (**232**) and epi-cyclonerodiol oxide (**233**) [50]. From another strain of *T. polysporum*, two antibiotic peptides, trichosporin Bs (**234**) [51] and trichosporin B-V (**235**) [52], were obtained.

### 2.15. Metabolites from Trichoderma reesei

Six compounds were isolated from the marine fungus *T. reesei*: cyclonerodiol (**92**); 8,9-dihydroxy-megastigmatrienone (**236**); harzialactone A (**237**); 3,6-dibenzylpiperazine-2,5-dione (**238**); 3-isobutyl-8-hydroxyl-pyrrolopiperazine-2,5-dione (**239**); and 3-benzyl-8-hydroxyl-pyrrolopiperazine-2,5-dione (**240**) [53].

### 2.16. Metabolites from Trichoderma saturnisporum

Fourteen compounds were isolated from *T. saturnisporum*: bislongiquinolide (**226**), cerebroside A (**241**); cerebroside D (**242**); sorbicillin A (**243**); sorbicillin B (**244**); bisvertinolone (**245**) [54]; and new sorbicillinoid-based saturnispols A–H (**246**–**253**) [55]. Among these, compounds **226**, **241**, **242**, and **245** showed the potential for antibacterial activity. Compound **251** exerted significant inhibition against a panel of bacteria strains, including vancomycin-resistant enterococci (VRE), with MIC ranging from 1.63 to 12.9 µg/mL, while compound **253** showed selective effects against VRE and *B. subtilis*.

### 2.17. Metabolites from Trichoderma spirale

Two compounds were isolated from the endophytic fungus *T. spirale* A17: tyrosol (**158**) and trichodemic acid (**254**). Compound **254** showed significant inhibitory activity against tumor cells SF-268, MCF-7, and NCI-H460, while compound **158** displayed weak hyperplasia inhibition activity against tumor cells [56].

### 2.18. Metabolites from Trichoderma virens

Four toxins were isolated from *T. viren*s ITC-4777: gliotoxin (**255**); dimethyl gliotoxin (**256**); viridin (**257**); and viridiol (**258**). Compound **255** was active against *Rhizoctonia bataticola* (with ED_50_ 0.03 µg/mL), *M. phaseolina* (with ED_50_ 1.76 µg/mL), *Pythium deharyanum* (with ED_50_ 29.38 µg/mL), *Pythium aphanidermatum* (with ED_50_ 12.02 µg/mL), *S. rolfsii* (with ED_50_ 2.11 µg/mL), and *R. solani* (with ED_50_ 3.18 µg/mL) [57].

Twenty-three compounds were identified from *T. virens* Y13-3: fourteen new compounds [trichorenins A–C (**259**–**261**); trichocarotins A–H (**262**–**269**); trichocadinin A (**270**); (3*S*,6*R*)-6-(para-hydroxybenzyl)-1,4-dimethyl-3,6-bis(methylthio)piperazine-2,5-dion (**271**); and dehydroxymethylbis(dethio)bis(methylthio)gliotoxin (**272**)] and nine known compounds [demethylincisterol A3 (**126**); CAF-603 (**273**); 14-hydroxy CAF-603 (**274**); 7-β-hydroxy CAF-603 (**275**); trichocaraneA(**276**); 3[(4′-hydroxyphenyl)methyl]-1,4-dimethyl-3,6-bis(methylthio)piperazine-2,5-dione (**277**); bis(dethio)bis(methylthio)gliotoxin (**278**); bisdethiobis(methylthio)-dehydrogliotoxin (**279**); and chromone (**280**)] [43]. Bioassays showed that compound **280** could remarkably inhibit *Pseudoalternaria citrea* with an IC_50_ value of 8 μg/mL; and compounds **270** and **276** showed potential brine shrimp lethality, with IC_50_ values of 17 and 21 μg/mL, respectively. In the experiment on growth inhibition of microalgae, compounds **259**–**261** had significant inhibitory effects on *C. marina* and *K. veneficum*, with IC_50_ values ranging from 0.41 to 1.0 μg/mL. Compounds **264**, **265**, **266**, **269** and **276** showed potent inhibitory activity against *C. marina*, *P. donghaiense, H. akashiwo*, and *K. veneficum*, with IC_50_ values ranging from 0.24 to 12 µg/mL.

### 2.19. Metabolites from Trichoderma viride

*T. viride* is widely used as a fungal antagonist. Twenty-eight compounds have been reported from *T. viride*: seventeen new antibiotic peptaibols [trichodecenins (**281**); trichorovins (**282**); trichocellins (**283**) [58]; and trichorovins I–XIV (**284**–**297**) [59]]; one new pyranone derivative, trichopyrone (**298**); and ten known compounds [bisvertinol (**76**); bislongiquinolide (**226**); trichodermanones A–D (**299**–**302**); rezishanone (**303**); vertinolide (**304**); trichodimerol (**305**); and 2-furancarboxylic acid (**306**)] [60].

### 2.20. Metabolites from Trichoderma viridescens

Two bioactive compounds were elucidated from *T. viridescens* TS0404: 6-pentyl-2*H*-pyran-2-one (**129**) and α-phenylcinnamic acid (**307**). Compound **129** had significant inhibitory activity against hyphal growth of *Phytophthora capsici*, *Phytophthora melonis*, *R. solani*, and *F. oxysporum* (with EC_50_ 115.26, 99.58, 126.46, and 315.75 µg/mL, respectively). The inhibitory effect on *P. melonis* was the best among them, and hyphal growth was completely inhibited when its concentration reached 300 µg/mL. Similarly, compound **129** had a conspicuous inhibitory effect on the zoosporangial germination of *P. capsici* and *P. melonis*, but the inhibitory effect on *P. melonis* was the most profound; and zoosporangial germination of *P. melonis* was completely inhibited at 400 µg/mL. In addition, compound **129** had a significant inhibitory effect on the conidial germination of *F. oxysporum* (with EC_50_ 151.81 µg/mL) and sclerotial germination of *R. solani* with complete inhibitory concentration 300 µg/mL [61].

### 2.21. Metabolites from Trichoderma spp.

A novel cyclopentenone, trichoderone (**308**), and a known compound, cholesta-7,22-diene-3β,5α,6β-triol (**309**), were identified from a marine *Trichoderma* sp. Compound **308** displayed potent cytotoxicity against A549, NCI-H460, MCF-7, MDA-MB-435s, HeLa-229, DU-145, and HLF. Compounds **308** and **309** also exhibited bioactivity against HIV protease and Taq DNA polymerase [62].

Four compounds were elucidated from mycelia of *Trichoderma* sp.: cyclonerodiol (**92**); 5α,8α-epidioxyergosta-6,22-diem-3β-ol (**310**); 1-monoolein (**311**); and methyl elaidate (**312**). Compound **92** showed weak nematicidal activity against *Panagrellus redivivus*, with 35.6% mortality at 800 mg/L in 72 h, and antimicrobial activity against *Paecilomyces lilacinus*, with an inhibition zone of 1.2 cm at 1 mg/disc [63].

One new compound, trichoderol A (**313**), was isolated from *Trichoderma* sp. cultures. Compound **313** was evaluated for antibacterial activity against *Pseudomonas putida*, *Nocardia brasiliensis*, and *Kocuria rhizophila*. The results showed compound **313** had antibacterial activity against the three pathogenic bacteria, with a MIC value of 5 μmol/L [64].

Two compounds were obtained from *Trichoderma* sp.: 6-pentyl-2*H*-pyran-2-one (**129**) and harzianic acid (**141**). Compounds **129** and **141** showed potential to improve plant growth and protect plant health [65].

Nine compounds were isolated from a sponge-derived *Trichoderma* sp. SCSIO41004: three new polyketides, [trichbenzoisochromen A (**314**); 5,7-dihydroxy-3-methyl-2-(2-oxopropyl)naphthalene-1,4-dione (**315**); and 7-acetyl-1,3,6-trihydroxyanthracene-9,10-dione (**316**)], and six known compounds [ZSU-H85 A (**317**); 1,3,6-trihydroxy-8-methytanthraquinone (**318**); 2,5-dimethyl-7-hydroxy-chromone (**319**); 7-hydroxy-2-(2′*S*-hydroxypropyl)-5-methylchromone (**320**); cyclonerotriol (**321**); and adenosine (**322**)] [66]. Compound **317** exhibited significant inhibitory activity against EV71 with an IC_50_ value of 25.7 μM.

Seventeen compounds were obtained from the endophytic fungus *Trichoderma* sp. 307 [64]: two new sesquiterpenes, microsphaeropsisins B (**323**) and C (**324**); two new de-o-methyllasiodiplodins, (3*R*,7*R*)-7-hydroxy-de-o-methyllasiodiplodin (**325**) and (3*R*)-5-oxo-de-o-methyllasiodiplodin (**326**); one new metabolite, (3*R*)-7-oxo-de-o-methyllasiodiplodin (**327**); and twelve known compounds [microsphaeropsisin (**328**); (3*R*)-5-oxolasiodiplodin (**329**); (3*S*)-6-oxo-de-o-methyllasiodiplodin (**330**); (3*R*)-de-o-methyllasiodiplodin (**331**); (3*R*,4*R*)-4-hydroxy-de-o-methyllasiodiplodin (**332**); (3*R*,5*R*)-5-hydroxy-de-o-methyllasiodiplodin (**333**); (3*R*,6*R*)-6-hydroxy-de-o-methyllasiodiplodin (**334**); (3*R*)-lasiodiplodin (**335**); (3*S*)-ozoroalide (**336**); (3*S*,5*R*)-5-hydroxylasiodiplodin (**337**); (*E*)-9-etheno-lasiodiplodin (**338**); and (3*R*)-nordinone (**339**). The isolated compounds were tested for their α-glucosidase inhibitory activity and cytotoxicity. Only compounds **325** and **326** exhibited potent α-glucosidase inhibitory activity with IC_50_ values of 25.8 and 54.6 µM, respectively [67].

An active antifungal compound, 2,5-cyclohexadiene-1,4-dione-2,6-bis(1,1-dimethylethyl) (**340**), was reported from *Trichoderma* sp. T-33 [68].

Three compounds were separated from *Trichoderma* sp. KK19L1: 5-hydroxy-3-hydroxymethyl-2-methyl-7-methoxychromone (**97**); (*E*)-3-acetylbenzylbut-2-enoate (**341**); and 1-hydroxy-6-methyl-9,10-anthraquinone (**342**)]. Compound **341** was a new compound [69].

Six compounds were isolated from *Trichoderma* sp. 09: methyl hexadecanoate (**343**); N-2′-hydroxy-3′*E*-octadecenoyl-1-o-β-D-glucopyranosyl-9-methyl-4*E*,8*E*-sphingadiene (**344**); (4*E*,8*E*)-1-o-(β-D-glucopyranosyl)-2-(2′-hydroxyl-(*E*)-3′-heptadecenoylamideo)-3-hydroxyl-9-methyl-4,8-nonadecadiene (**345**); ergosta-7,24(28)-diene-3β-ol (**346**); cholest-4-ene-3-ol (**347**); and methyl decanoate (**348**). Primary bioassay showed that compound **344** exhibited moderate inhibitory activity against *Fusarium graminearum*, *Calletotrichum musae*, and *Penicillium italicum;* and compound **345** exhibited moderate inhibitory activity against *F. graminearum* and *C. musae* and low inhibitory activity against *P. italicum* at a concentration of 0.5 μmol/mL [70].

Two unusual pyridines, trichodins A (**349**) and B (**350**), together with a known compound, pyridoxatin (**351**), were extracted from the marine *Trichoderma* sp. MF106. Compounds **349** and **351** showed antibiotic activities against the clinically relevant microorganism *Staphylococcus epidermidis*, with IC_50_ values of 24 μM and 4 μM, respectively [71].

A nematicidal compound, trichodermin (**70**), was isolated from the ethyl acetate extract of *Trichoderma* sp. YMF1.02647. Compound **70** killed more than 95% of both *Panagrellus redivivus* and *Caenorhabditis elegans* in 72 h at 0.4 g/L [72].

Two new cyclopentenones, trichodermones A (**47**) and B (**352**), together with a known compound, 3-(3-oxocyclopent-1-enyl)propanoic acid (**353**), were obtained from *Trichoderma* sp. YLF-3. These compounds were assayed for antibacterial activity, and compound **353** showed activity against *Staphyloccocus aureus* and *Bacillus cereus* [73].

Two novel compounds were isolated from *Trichoderma* sp. USF-2690: demethylsorbicillin (**354**) and oxosorbicillinol (**355**). In a 1,1-diphenyl-2-picrylhydrazyl (DPPH) radical-scavenging asssy, compound **355** gave an ED_50_ value of 87.7 µM [74].

Thirteen compounds were obtained from the fermentation broth of *Trichoderma* sp. Jing-8: a new natural mycotoxin, alternariol 1′-hydroxy-9-methyl ether (**356**), and twelve known compounds [ergosterol (**134**); and cerevisterol (**221**); alternariol 9-methyl ether (**357**); alternariol (**358**); altechromone A (**359**); altenuene (**360**); 4′-epialtenuene (**361**); scytalone (**362**); α-acetylorcinol (**363**); cerebroside C (**364**); α-palmitoyl-β-linoleoyl-α′-linoleoyl glycerol (**365**); and 1,2-benzenedicarboxylic acid bis(2*S*-methyl heptyl) ester (**366**)]. Compounds **356**, **363,** and **364** showed an inhibitory effect against cabbage seed germination (MIC < 3 μg/mL). Compound **356** showed antibacterial activity against *B. subtilis* and *S. aureus* (with MIC 64 μg/mL). Compounds **356** and **358** showed significant DPPH radical-scavenging activity (with IC_50_ 12 μg/mL) [75].

Eight known compounds were isolated from *Trichoderma* sp. TA26-28: nafuredin (**94**); 5-hydroxy-2,3-dimethyl-7-methoxychromone (**96**); cerebroside D (**242**); cerebroside C (**364**); pachybasin (**367**); chrysophanol (**368**); 8-*o*-methylchrysophanol (**369**); and soya-cerebroside I (**370**). In the research, MIC (µM) values of eight compounds were evaluated against a panel of pathogenic bacteria: six Gram-positive bacteria [*S. aureus*, *Sardine albus*, *B. cereus*, *B. subtilis*, *Micrococcus tetragenus*, and *K. rhizophila*] and four Gram-negative bacteria [*E. coli*, *V. parahaemolyticus*, *V. anguillarum*, and *P. putida*]. Compound **96** showed pronounced antibacterial activity against all the tested bacteria, with MIC values ranging from 0.78 to 6.25 M. In addition, compounds **242** and **364** showed selective antibacterial activity against Gram-negative bacteria, and compound **94** showed weak antibacterial activity against *B. cereus* and *P. putida* [76].

Nine compounds were obtained from *Trichoderma* sp. YM311505: 3β,5α,9α-trihydroxyergosta-7,22-dien-6-one (**31**); ergosterol (**134**); trichodimerol (**305**); 5α,6α-epoxyergosta-8(14),22-diene-3β,7α-diol (**371**); campesterol (**372**); 7-methoxy-4,6-dimethyl phthalide (**373**); 7-hydroxy-4,6-dimethyl phtalide (**374**); daidzein (**375**); and cinnamic acid (**376**). Compound **31** exhibited the most potent antifungal activities against *P. oryzae*, *C. albicans*, *Aspergillus niger*, and *Alternaria alternata* with MIC value at 32 µg/mL. Compound **373** showed antimicrobial activity against *E. coli*, *B. subtilis*, *P. oryzae*, *A. niger* and *A. alternata* with MIC 64 µg/mL. Compounds **373** and **375** exhibited antibacterial activity against *E. coli* with MIC 64 µg/mL. Compound **305** showed antifungal activity against *P. oryzae*, *C. albicans*, and *A. niger* with MIC values of 32, 32, and 64 µg/mL, respectively [77].

Seventeen compounds were isolated from the endophytic fungus *Trichoderma* sp. Xy24: cyclonerodiol (**92**); ergosterol (**134**); trichodimerol (**305**); trichoacorenol (**377**) [78]; trichocage B (**378**); 1α-isopropyl-4α,8-dimethylspirod[4.5]-dec8-ene-2β,7α-di-ol (**379**); 1α-isopropyl-4α,8-dimethyl-spiro[4.5]dec-8-ene-3β,7α-diol (**380**); 10,11-dihydroxy-cyclonerodiol (**381**); 14-hydroxy-trichoacorenol (**382**); harzianone (**383**); (9*R*,10*R*)-dihydro-harzianone (**384**); ergokonin B (**385**); methyl stearate (**386**) [79]; harzianelactone (**387**); trichoacorenol B (**388**); trichoacorenol C (**389**); and cyclonerodiol B (**390**) [80]. Among them, compounds **381**, **382**, and **384** were new. Compound **305** exhibited medium inhibitory activity (with IC_50_ 74.6 μM), using a neuraminidase (H7N9)/methylumbelliferyl-N-acetylneuraminic acid model. Compound **384** showed cytotoxic activity against the HeLa with IC_50_ 30.1 μM and MCF-7 cell line with IC_50_ 30.7 μM. Compound **390** inhibited LPS-induced NO production in BV2 cells by 75.0% (0.1 µM) and had good neuro-anti-inflammatory activity.

All secondary metabolites from *Trichoderma* are summarized in Table 1.

## 3. Conclusions

*Trichoderma* species are known for their diverse bioactivity owing to the production of abundant secondary metabolites. Hundreds of metabolites produced by *Trichoderma* have been isolated and characterized. In this review, 390 non-volatile compounds from 20 known species and various *Trichoderma* spp. were summarized. These compounds included peptaibols, terpenes, diketopiperazines, steroids, amides, lactones, polyketides, tetronic acid derivatives, peptides, pyranone derivatives, pyridines, and cyclopentenones. These compounds exhibited numerous biological activities, including cytotoxic, anti-tumor, antifungal, antibacterial, antiviral, antibiotic, anti-inflammatory, antimicroalgal, plant-growth-enhancing/inhibitoring, bioinducer, hyperplasia inhibitory, siderophoric, antagonism, nematicidal, plant resistance, DPPH radical scavenging, and enzyme inhibitory effects.

Some metabolites were found in different species of *Trichoderma*. The antifungal and nematicidal compound trichodermin (**70**) was found in *T. brevicompactum*, *T. harzianum*, and *Trichoderma* sp. YMF1.02647. The bioactive metabolite 6-pentyl-α-pyrone (**67**) was distributed both in *T. atroviride* and *T. harzianum*. Cyclonerodiol (**92**) was found in *T. citrinoviride*, *T. harzianum*, *T. koningüi*, *T. reesei*, and *Trichoderma* sp. Lignoren (**64**) was obtained from three species (*T. atroviride*, *T. citrinoviride*, and *T. lignorum)* and showed antimicrobial activity. Numerous strains from different species of *Trichoderma* had the same bioactivity, perhaps due to their identical metabolites.

Although *Trichoderma* spp. have been widely studied, more metabolites will likely be identified in the future.

## Figures and Tables

**Table 1:** Non-volatile metabolites and their biological activities from *Trichoderma.*

Metabolites	Species	Activity	Refs.	Metabolites	Species	Activity	Refs.
prealamethicin F50 (**1**)	*T. arundinaceum*	-	[3]	trikoningin KB I (**196**)	*T. koningiopsis*	-	[1]
Glu(OMe)^18^-alamethicin F50 (**2**)	*T. arundinaceum*	Anti-tumor	[3]	trikoningin KB II (**197**)	*T. koningiopsis*	-	[1]
trichobrevin BIII-D (**3**)	*T. arundinaceum*	Anti-tumor	[3]	konginginin A (**198**)	*T. koningiopsis* YIM PH30002	Siderophoric Antifungal Antibacterial	[42]
alamethicin F50 (**4**)	*T. arundinaceum*	-	[3]	konginginin B (**199**)	*T. koningiopsis* YIM PH30002	Siderophoric Antifungal Antibacterial	[42]
alamethicin II (**5**)	*T. arundinaceum*	-	[3]	konginginin D (**200**)	*T. koningiopsis* YIM PH30002	Siderophoric Antifungal Antibacterial	[42]
atroviridin J (**6**)	*T. arundinaceum*	-	[3]	konginginin F (**201**)	*T. koningiopsis* YIM PH30002	Siderophoric Antifungal Antibacterial	[42]
trichobranchin D-I (**7**)	*T. arundinaceum*	-	[3]	konginginin M (**202**)	*T. koningiopsis* YIM PH30002	Antifungal Antibacterial	[42]
trichodermaerin (**8**)	*T. asperellum*	-	[5]	3-acetyl-6-methyl-2*H*-pyran-2,4(3*H*)-dione (**203**)	*T. koningiopsis* Y10-2	-	[43]
6-amyl alpha-pyrone (**9**)	*T. asperellum*	-	[6]	lutidonecarboxylic acid (**204**)	*T. koningiopsis* Y10-2	-	[43]
aspereline G (**10**)	*T. asperellum*	-	[7]	cyclonertriol (**205**)	*T. koningiopsis* Y10-2	-	[43]
aspereline H (**11**)	*T. asperellum*	-	[7]	2-hydroxydiplopterol (**206**)	*T. koningiopsis* Y10-2	-	[43]
aspereline A (**12**)	*T. asperellum*	-	[7]	verrucosidin (**207**)	*T. koningiopsis* Y10-2	-	[43]
aspereline C (**13**)	*T. asperellum*	-	[7]	neoechinulin A (**208**)	*T. koningiopsis* Y10-2	-	[43]
aspereline D (**14**)	*T. asperellum*	-	[7]	isoechinulin A (**209**)	*T. koningiopsis* Y10-2	Antimicroalgal	[43]
aspereline E (**15**)	*T. asperellum*	-	[7]	echinuline (**210**)	*T. koningiopsis* Y10-2	Antimicroalgal	[43]
aspereline F (**16**)	*T. asperellum*	-	[7]	cyclo-trans-4-OH-(D)-Pro-(D)-Phe (**211**)	*T. koningiopsis* Y10-2	-	[43]
bisabolan-1,10,11-triol (**17**)	*T. asperellum* cf44-2	Antibacterial Growth inhibitoring	[8]	fructigenine A (**212**)	*T. koningiopsis* Y10-2	Antimicroalgal	[43]
12-nor-11-acetoxybisabolen-3,6,7-triol (**18**)	*T. asperellum* cf44-2	Antibacterial Growth inhibitoring	[8]	3-*o*-methylviridicatin (**213**)	*T. koningiopsis* Y10-2	-	[43]
(7*S*)-1-hydroxy-3-*p*-menthen-9-oic acid (**19**)	*T. asperellum* cf44-2	-	[8]	cyclopenol (**214**)	*T. koningiopsis* Y10-2	Antibacterial	[43]
(7*R*)-1-hydroxy-3-*p*-menthen-9-oic acid (**20**)	*T. asperellum* cf44-2	-	[8]	olemolide (**215**)	*T. koningiopsis* Y10-2	-	[43]
dechlorotrichodenone C (**21**)	*T. asperellum* cf44-2	Antibacterial Growth inhibitoring	[8]	4-hydroxyphenylacetate (**216**)	*T. koningiopsis* Y10-2	-	[43]
3-hydroxytrichodenone C (**22**)	*T. asperellum* cf44-2	Antibacterial Growth inhibitoring	[8]	4-hydroxyphenylethanol (**217**)	*T. koningiopsis* Y10-2	-	[43]
methylcordysinin A (**23**)	*T. asperellum* cf44-2	-	[8]	m-methoxyphenol (**218**)	*T. koningiopsis* Y10-2	-	[43]
4-oxazolepropanoic acid (**24**)	*T. asperellum* cf44-2	-	[8]	sorbicillin (**219**)	*T. longibrachiatum*	Antibacterial	[45]
wickerol A (**25**)	*T. asperellum* dl-34 *T. koningiopsis* Y10-2	Nematicidal	[9] [43]	ergosterol peroxide (**220**)	*T. longibrachiatum*	-	[45]
harziandione (**26**)	*T. asperellum* dl-34 *T. koningiopsis* Y10-2	Nematicidal	[9] [43]	cerevisterol (**221**)	*T. longibrachiatum*	-	[45] [75]
ergosterol endoperoxide (**27**)	*T. asperellum* dl-34 *T. citrinoviride* cf-27 *T. harzianum* R5	-	[9]	2-anhydromevalonic acid (**222**)	*T. longibrachiatum*	-	[45]
5α,8α-epidioxyergosta-6,9(11),22-trien-3β-ol (**28**)	*T. asperellum* dl-34 *T. harzianum* R5 *T. harzianum* dl-36	-	[9] [24]	squalene (**223**)	*T. longibrachiatum*	-	[45]
3β,5α,6β-trihydroxyergosta-7,22-diene (**29**)	*T. asperellum* dl-34 *T. harzianum* R5	-	[9]	ergokonin A (**224**)	*T. longibrachiatum*	Antifungal	[46]
3β,5α-dihydroxy-6β-methoxyergosta-7,22-diene (**30**)	*T. asperellum* dl-34	-	[9]	5-hydroxyvertinolide (**225**)	*T. longibrachiatum* Rifai	Antagonism	[47]
3β,5α,9α-trihydroxyergosta-7,22-dien-6-one (**31**)	*T. asperellum* dl-34 *T. harzianum* dl-36 *Trichoderma* sp. YM311505	Antifungal	[9] [24] [77]	bislongiquinolide (**226**)	*T. longibrachiatum* Rifai *T. saturnisporum* *T. viride*	Antagonism Antibacterial	[47] [54] [60]
(22*E*,24*R*)-ergosta-4,6,8,(14),22-tetraen-3-one (**32**)	*T. asperellum* dl-34 *T. citrinoviride* cf-27	-	[9]	10,11-dihydrocyclonerotriol (**227**)	*T. longibrachiatum* YM311505	Antifungal	[48]
(22*E*,24*R*)-5α,6α-epoxyergosta-8,22-diene-3β,7α-diol (**33**)	*T. asperellum* dl-34	-	[9]	sohirnone A (**228**)	*T. longibrachiatum* YM311505	Antifungal	[48]
ergosta-7,22-dien-3β-ol (**34**)	*T. asperellum* dl-34	-	[9]	trichokonin A (**229**)	*T. longibrachiatum* SMF2	Antiviral Anti-tumor Antimicrobial Plant resistance	[49]
(22*E*,24*R*)-ergosta-5,7,22-trien-3β-ol (**35**)	*T. asperellum* dl-34 *T. citrinoviride* cf-27 *T. harzianum* dl-36	-	[9] [24]	trichokonin B (**230**)	*T. longibrachiatum* SMF2	-	[49]
β-sitosterol (**36**)	*T. asperellum* dl-34 *T. harzianum* T-4 *T. longibrachiatum*	-	[9] [22] [33]	valinotricin (**231**)	*T. polysporum*	-	[50]
(L)-Pro-(L)-Leu (**37**)	*T. asperellum* dl-34	-	[9]	cyclonerodiol oxide (**232**)	*T. polysporum*	-	[50]
(L)-4-OH-Pro-(L)-Leu (**38**)	*T. asperellum* dl-34	-	[9]	epi-cyclonerodiol oxide (**233**)	*T. polysporum*	-	[50]
adenine nucleoside (**39**)	*T. asperellum* dl-34 *T. harzianum* R5	-	[9]	trichosporin Bs (**234**)	*T. polysporum*	-	[51]
cis-4-hydroxy-6-deoxyscytalone (**40**)	*T. asperellum* dl-34	-	[9]	trichosporin B-V (**235**)	*T. polysporum*	-	[52]
2,4-dihydroxy-3,6-dimethylbenzaldehyde (**41**)	*T. asperellum* dl-34	-	[9]	8,9-dihydroxy-megastigmatrienone (**236**)	*T. reesei*	-	[53]
dihydrocitrinone (**42**)	*T. asperellum* dl-34	-	[9]	harzialactone A (**237**)	*T. reesei*	-	[53]
atrichodermone A (**43**)	*T. atroviride*	Cytotoxic Anti-inflammatory	[10]	3,6-dibenzylpiperazine-2,5-dione (**238**)	*T. reesei*	-	[53]
atrichodermone B (**44**)	*T. atroviride*	Cytotoxic Anti-inflammatory	[10]	3-isobutyl-8-hydroxyl-pyrrolopiperazine-2,5-dione (**239**)	*T. reesei*	-	[53]
atrichodermone C (**45**)	*T. atroviride*	Cytotoxic Anti-inflammatory	[10]	3-benzyl-8-hydroxyl-pyrrolopiperazine-2,5-dione (**240**)	*T. reesei*	-	[53]
atrichodermone D (**46**)	*T. atroviride*	-	[11]	cerebroside A (**241**)	*T. saturnisporum*	Antibacterial	[54]
trichodermone A (**47**)	*T. atroviride* *Trichoderma* sp. YLF-3	-	[11] [73]	cerebroside D (**242**)	*T. saturnisporum* *Trichoderma* sp. TA26-28	Antibacterial	[54] [76]
(5*R*)5-hydroxy-3-[(methoxycarbonyl)-amino]-5-vinyl-2-cyclopenten-1-one (**48**)	*T. atroviride*	-	[11]	sorbicillin A (**243**)	*T. saturnisporum*	-	[54]
4*H*-1,3-dioxin-4-one-2,3,6-trimethyl (**49**)	*T. atroviride*	Antibacterial Cytotoxic	[11]	sorbicillin B (**244**)	*T. saturnisporum*	-	[54]
1,3-dione-5,5-dimethylcyclohexane (**50**)	*T. atroviride* *T. harzianum*	-	[11]	bisvertinolone (**245**)	*T. saturnisporum*	Antibacterial	[54]
2-enone-3hydroxy-5,5-dimethylcylohex (**51**)	*T. atroviride*	-	[11]	saturnispol A (**246**)	*T. saturnisporum*	-	[55]
6-pentyl-pyran-2-one (**52**)	*T. atroviride*	-	[1] [25] [26]	saturnispol B (**247**)	*T. saturnisporum*	-	[55]
6-pent-1-enyl-pyrane-2-one (**53**)	*T. atroviride*	-	[1]	saturnispol C (**248**)	*T. saturnisporum*	-	[55]
2-hydroxybutan-3-yl5′-(2″-hydroxy-N-(2‴-oxobutan-3‴-yl)propanamido)butanoate (**54**)	*T. atroviride* G20-12	-	[12]	saturnispol D (**249**)	*T. saturnisporum*	-	[55]
3-hydroxy-5-(4-hydroxybenzyl)dihydrofuran-2(3*H*)-one (**55**)	*T. atroviride* G20-12	-	[12]	saturnispol E (**250**)	*T. saturnisporum*	-	[55]
4′-(4,5-dimethyl-1,3-dioxolan-2-yl)methyl-phenol (**56**)	*T. atroviride* G20-12	-	[13]	saturnispol F (**251**)	*T. saturnisporum*	-	[55]
(3′-hydroxybutan-2′-yl)5-oxopyrrolidine-2-carboxylate (**57**)	*T. atroviride* G20-12	-	[13]	saturnispol G (**252**)	*T. saturnisporum*	-	[55]
atroviridetide (**58**)	*T. atroviride* G20-12	-	[13]	saturnispol H (**253**)	*T. saturnisporum*	Antibacterial	[55]
trichodermadione A (**59**)	*T. atroviride* S361	-	[14]	trichodemic acid (**254**)	*T. spirale* A17	Anti-tumor	[56]
trichodermadione B (**60**)	*T. atroviride* S361	-	[14]	gliotoxin (**255**)	*T. viren*s ITC-4777	Antifungal	[57]
4-(2-formyl-5-(methoxymethyl)-1*H*-pyrrol-1-yl)butanoic acid (**61**)	*T. atroviride* S361	-	[14]	dimethyl gliotoxin (**256**)	*T. viren*s ITC-4777	-	[57]
5-methoxymethyl-1*H*-pyrrole-2-carbaalde-hyde (**62**)	*T. atroviride* S361	-	[14]	viridin (**257**)	*T. viren*s ITC-4777	-	[57]
3-(1-carbaalde)-6-methyl-2*H*-pyran-2,4(3*H*)-dione (**63**)	*T. atroviride* S361	-	[14]	viridiol (**258**)	*T. viren*s ITC-4777	-	[57]
lignoren (**64**)	*T. atroviride* S361 *T. citrinoviride* *T. lignorum* HKI 0257	Antibacterial	[14], [18], [44]	trichorenin A (**259**)	*T. virens* Y13-3	Antimicroalgal	[43]
ascotrichic acid (**65**)	*T. atroviride* S361	-	[14]	trichorenin B (**260**)	*T. virens* Y13-3	Antimicroalgal	[43]
catenioblin C (**66**)	*T. atroviride* S361 *T. longibrachiatum* YM311505	Antifungal	[14]	trichorenin C (**261**)	*T. virens* Y13-3	Antimicroalgal	[43]
6-pentyl-α-pyrone (**67**)	*T. atroviride* UST1 *T. atroviride* UST2 *T. harzianum* T77 *T. harzianum* SQR-T037	Plant resistance Antifungal	[15] [35]	trichocarotin A (**262**)	*T. virens* Y13-3	-	[43]
koninginin G (**68**)	*T. aureoviride*	Growth inhibitoring	[16]	trichocarotin B (**263**)	*T. virens* Y13-3	-	[43]
Koninginin G triacetate (**69**)	*T. aureoviride*	-	[16]	trichocarotin C (**264**)	*T. virens* Y13-3	Antimicroalgal	[43]
trichodermin (**70**)	*T. brevicompactum* *T. harzianum* *Trichoderma* sp.YMF1.02647	Antifungal Nematicidal	[17] [25] [26] [72]	trichocarotin D (**265**)	*T. virens* Y13-3	Antimicroalgal	[43]
(*R*)-vertinolide (**71**)	*T. citrinoviride*	-	[1]	trichocarotin E (**266**)	*T. virens* Y13-3	Antimicroalgal	[43]
trichoderiol C (**72**)	*T. citrinoviride*	-	[18]	trichocarotin F (**267**)	*T. virens* Y13-3	-	[43]
citrinoviric acid (**73**)	*T. citrinoviride*	Cytotoxic	[18]	trichocarotin G (**268**)	*T. virens* Y13-3	-	[43]
penicillenol D (**74**)	*T. citrinoviride*	Cytotoxic	[18]	trichocarotin H (**269**)	*T. virens* Y13-3	Antimicroalgal	[43]
trichotetronine (**75**)	*T. citrinoviride*	-	[18]	trichocadinin A (**270**)	*T. virens* Y13-3	-	[43]
bisvertinol (**76**)	*T. citrinoviride* *T. viride*	-	[6] [18]	(3*S*,6*R*)-6-(para-hydroxybenzyl)-1,4-dimethyl-3,6-bis(methylthio)piperazine-2,5-dion (**271**)	*T. virens* Y13-3	-	[43]
spirosorbicillinol A (**77**)	*T. citrinoviride*	-	[18]	dehydroxymethylbis(dethio)bis(methylthio)gliotoxin (**272**)	*T. virens* Y13-3	-	[43]
spirosorbicillinol B (**78**)	*T. citrinoviride*	-	[18]	CAF-603 (**273**)	*T. virens* Y13-3	-	[43]
spirosorbicillinol C (**79**)	*T. citrinoviride*	-	[18]	14-hydroxy CAF-603 (**274**)	*T. virens* Y13-3	-	[43]
trichoderiol A (**80**)	*T. citrinoviride*	-	[18]	7-β-hydroxy CAF-603 (**275**)	*T. virens* Y13-3	-	[43]
penicillenol B_1_ (**81**)	*T. citrinoviride*	-	[18]	trichocarane A(**276**)	*T. virens* Y13-3	Antimicroalgal	[43]
penicillenol B_2_ (**82**)	*T. citrinoviride*	-	[18]	3[(4′-hydroxyphenyl)methyl]-1,4-dimethyl-3,6-bis(methylthio)piperazine-2,5-dione (**277**)	*T. virens* Y13-3	-	[43]
cyclo-(Leu-Pro) (**83**)	*T. citrinoviride*	-	[18]	bis(dethio)bis(methylthio)gliotoxin (**278**)	*T. virens* Y13-3	-	[43]
cyclo-(Ile-Pro) (**84**)	*T. citrinoviride*	-	[18]	bisdethiobis(methylthio)-dehydrogliotoxin (**279**)	*T. virens* Y13-3	-	[43]
cyclo-(Phe-Pro) (**85**)	*T. citrinoviride*	-	[18]	chromone (**280**)	*T. virens* Y13-3	Antifungal	[43]
trichocitrin (**86**)	*T. citrinoviride* cf-27	Antimicroalgal	[9]	trichodecenins (**281**)	*T. viride*	-	[58]
24-methylenecycloartanol (**87**)	*T. citrinoviride* cf-27	-	[9]	trichorovins (**282**)	*T. viride*	-	[58]
cycloeucalenol (**88**)	*T. citrinoviride* cf-27	-	[9]	trichocellins (**283**)	*T. viride*	-	[58]
citrostadienol (**89**)	*T. citrinoviride* cf-27	-	[9]	trichorovin I (**284**)	*T. viride*	-	[59]
euphorbol (**90**)	*T. citrinoviride* cf-27	-	[9]	trichorovin II (**285**)	*T. viride*	-	[59]
24-methylene-lanost-8-en-3β-ol (**91**)	*T. citrinoviride* cf-27	-	[9]	trichorovin III (**286**)	*T. viride*	-	[59]
cyclonerodiol (**92**)	*T. citrinoviride* cf-27 *T. harzianum* R5-1 *T. harzianum* *T. koningiopsis* Y10-2 *T. reesei* *Trichoderma* sp *Trichoderma* sp. Xy24	Antibacterial Antifungal Nematicidal	[9] [21] [27] [43] [53] [63] [78]	trichorovin IV (**287**)	*T. viride*	-	[59]
(22*E*,24*R*)-7β,8β-epoxy-3β,5α,9α-trihydroxyergosta-22-en-6-one (**93**)	*T. citrinoviride* cf-27	-	[9]	trichorovin V (**288**)	*T. viride*	-	[59]
nafuredin (**94**)	*T. citrinoviride* cf-27 *Trichoderma* sp. TA26-28	Antimicroalgal Antibacterial	[9] [76]	trichorovin VI (**289**)	*T. viride*	-	[59]
harzianolide (**95**)	*T. citrinoviride* cf-27 *T. harzianum* T22 *T. harzianum* T39 *T. harzianum* dl-36	Antibacterial Antifungal	[9] [23] [24]	trichorovin VII (**290**)	*T. viride*	-	[59]
5-hydroxy-2,3-dimethyl-7-methoxychromone (**96**)	*T. citrinoviride* cf-27 *Trichoderma* sp. TA26-28	Antibacterial	[9] [76]	trichorovin VIII (**291**)	*T. viride*	-	[59]
5-hydroxy-3-hydroxymethyl-2-methyl-7-methoxychromone (**97**)	*T. citrinoviride* cf-27 *Trichoderma* sp. KK19L1	-	[9] [69]	trichorovin IX (**292**)	*T. viride*	-	[59]
methyl 8-hydroxy-6-methyl-9-oxo-9*H*-xanthene-1-carboxylate (**98**)	*T. citrinoviride* cf-27	-	[9]	trichorovin X (**293**)	*T. viride*	-	[59]
methyl 2,8-dihydroxy-6-methyl-9-oxo-9*H*-xanthene-1-carboxylate (**99**)	*T. citrinoviride* cf-27	-	[9]	trichorovin XI (**294**)	*T. viride*	-	[59]
stachyline B (**100**)	*T. citrinoviride* cf-27	-	[9]	trichorovin XII (**295**)	*T. viride*	-	[59]
trans-3,4-dihydro-2,4,8-trihydroxynaphthalen-1(2*H*)-one (**101**)	*T. citrinoviride* cf-27	-	[9]	trichorovin XIII (**296**)	*T. viride*	-	[59]
pyrazole-3-carboxylic acid (**102**)	*T. citrinoviride* cf-27	-	[9]	trichorovin XIV (**297**)	*T. viride*	-	[59]
pyrrole-2-carboxylic acid (**103**)	*T. citrinoviride* cf-27	-	[9]	trichopyrone (**298**)	*T. viride*	-	[60]
dibutyl phthalate (**104**)	*T. citrinoviride* cf-27	-	[9]	trichodermanone A (**299**)	*T. viride*	-	[60]
cremenolide (**105**)	*T. cremeum*	Antifungal Growth enhancing	[19]	trichodermanone B (**300**)	*T. viride*	-	[60]
trichoderone A (**106**)	*T. gamsii*	-	[20]	trichodermanone C (**301**)	*T. viride*	-	[60]
trichoderone B (**107**)	*T. gamsii*	-	[20]	trichodermanone D (**302**)	*T. viride*	-	[60]
aspochalasin D (**108**)	*T. gamsii*	Cytotoxic	[20]	rezishanone (**303**)	*T. viride*	-	[60]
aspochalasin J (**109**)	*T. gamsii*	Cytotoxic	[20]	vertinolide (**304**)	*T. viride*	-	[60]
aspochalasin I (**110**)	*T. gamsii*	-	[20]	trichodimerol (**305**)	*T. viride* *Trichoderma* sp. YM311505 *Trichoderma* sp. Xy24	Antifungal Enzyme inhibitoring	[60] [77] [78]
trichoharzianin (**111**)	*T. harzianum* R5	Antimicroalgal	[9]	2-furancarboxylic acid (**306**)	*T. viride*	-	[60]
3β-hydroxyergosta-8,24(28)-dien-7-one (**112**)	*T. harzianum* R5	-	[9]	α-phenylcinnamic acid (**307**)	*T. viridescens* TS0404	-	[61]
(22*E*,24*R*)-24-methylcholesta-5,22-dien-3β-ol (**113**)	*T. harzianum* R5	-	[9]	trichoderone (**308**)	*Trichoderma* sp	Cytotoxic Enzyme inhibitoring	[62]
5,7-dihydroxy-2,3-dimethylchromone (**114**)	*T. harzianum* R5	-	[9]	cholesta-7,22-diene-3β,5α,6β-triol (**309**)	*Trichoderma* sp	Enzyme inhibitoring	[62]
(22*E*,24*R*)-3β,5α-dihydroxy-ergosta-7,22-dien-6-one (**115**)	*T. harzianum* R5	-	[9]	5α,8α-epidioxyergosta-6,22-diem-3β-ol (**310**)	*Trichoderma* sp	-	[63]
5-hydroxy-2-hydroxymethyl-3-methyl-7-methoxychromone (**116**)	*T. harzianum* R5	-	[9]	1-monoolein (**311**)	*Trichoderma* sp	-	[63]
indole-3-carboxaldehyde (**117**)	*T. harzianum* R5	-	[9]	methyl elaidate (**312**)	*Trichoderma* sp	-	[63]
3-indol acetic acid (**118**)	*T. harzianum* R5	-	[9]	trichoderol A (**313**)	*Trichoderma* sp	Antibacterial	[64]
2,4-dimethylbenzene-1,3,5-triol (**119**)	*T. harzianum* R5	-	[9]	trichbenzoisochromen A (**314**)	*Trichoderma* sp. SCSIO41004	-	[66]
5′-*o*-acetyluracil nucleoside (**120**)	*T. harzianum* R5	-	[9]	5,7-dihydroxy-3-methyl-2-(2-oxopropyl)naphthalene-1,4-dione (**315**)	*Trichoderma* sp. SCSIO41004	-	[66]
wickerol B (**121**)	*T. harzianum* R5-1 *T. koningiopsis* Y10-2	Antibacterial	[21] [43]	7-acetyl-1,3,6-trihydroxyanthracene-9,10-dione (**316**)	*Trichoderma* sp. SCSIO41004	-	[66]
(1*S*,4*S*,5*S*)-8-hydroxymethyl-1-isopropyl-4-methylspiro[4.5]dec-8-en-7-one) (**122**)	*T. harzianum* R5-1	Antibacterial	[21]	ZSU-H85 A (**317**)	*Trichoderma* sp. SCSIO41004	Antiviral	[66]
epicycloneodiol oxide (**123**)	*T. harzianum* R5-1 *T. koningiopsis* Y10-2	Antibacterial	[21] [43]	1,3,6-trihydroxy-8-methytanthraquinone (**318**)	*Trichoderma* sp. SCSIO41004	-	[66]
cycloneodiol oxide (**124**)	*T. harzianum* R5-1 *T. koningiopsis* Y10-2	Antibacterial	[21] [43]	2,5-dimethyl-7-hydroxy-chromone (**319**)	*Trichoderma* sp. SCSIO41004	-	[66]
5,6-dihydro-4-methyl-2*H*-pyran-2-one (**125**)	*T. harzianum* R5-1	-	[21]	7-hydroxy-2-(2′*S*-hydroxypropyl)-5-methylchromone (**320**)	*Trichoderma* sp. SCSIO41004	-	[66]
demethylincisterol A3 (**126**)	*T. harzianum* R5-1 *T. virens* Y13-3	-	[21] [43]	cyclonerotriol (**321**)	*Trichoderma* sp. SCSIO41004	-	[66]
palmitic acid (**127**)	*T. harzianum* T-4 *T. koningii* T-8	-	[22] [38]	adenosine (**322**)	*Trichoderma* sp. SCSIO41004	-	[66]
1,8-dihydroxy-3-methylanthraquinone (**128**)	*T. harzianum* T-4 *T. harzianum* T22 *T. harzianum* T39	-	[22] [23]	microsphaeropsisin B (**323**)	*Trichoderma* sp. 307	-	[64]
6-pentyl-2*H*-pyran-2-one (**129**)	*T. harzianum* T-4 *T. viridescens* TS0404 *Trichoderma* sp	Antifungal Growth inhibitoring	[22] [61] [65]	microsphaeropsisin C (**324**)	*Trichoderma* sp. 307	-	[64]
2(5*H*)-furanone (**130**)	*T. harzianum* T-4	-	[22]	(3*R*,7*R*)-7-hydroxy-de-o-methyllasiodiplodin (**325**)	*Trichoderma* sp. 307	Enzyme inhibitoring	[64] [67]
stigmasterol (**131**)	*T. harzianum* T-4 *T. koningii* T-11	-	[22] [38]	(3*R*)-5-oxo-de-o-methyllasiodiplodin (**326**)	*Trichoderma* sp. 307	Enzyme inhibitoring	[64] [67]
1-hydroxy-3-methylanthraquinone (**132**)	*T. harzianum* T-4 *T. harzianum* T22 *T. harzianum* T39	-	[22] [23]	(3*R*)-7-oxo-de-o-methyllasiodiplodin (**327**)	*Trichoderma* sp. 307	-	[64]
δ-decanolactone (**133**)	*T. harzianum* T-4 *T. koningii* T-8	-	[22] [38]	microsphaeropsisin (**328**)	*Trichoderma* sp. 307	-	[64]
ergosterol (**134**)	*T. harzianum* T-4 *T. longibrachiatum* *Trichoderma* sp. YM311505 *Trichoderma* sp. Xy24	-	[22] [33] [75] [77] [78]	(3*R*)-5-oxolasiodiplodin (**329**)	*Trichoderma* sp. 307	-	[64]
harzianopyridone (**135**)	*T. harzianum* T-4 *T. harzianum* T22 *T. harzianum* T39	Antifungal	[22] [23]	(3*S*)-6-oxo-de-o-methyllasiodiplodin (**330**)	*Trichoderma* sp. 307	-	[64]
6-methyl-1,3,8-trihydroxyanthraquinone (**136**)	*T. harzianum* T-4	-	[22]	(3*R*)-de-o-methyllasiodiplodin (**331**)	*Trichoderma* sp. 307	-	[64]
T22azaphilone (**137**)	*T. harzianum* T22 *T. harzianum* T39	Antifungal	[23]	(3*R*,4*R*)-4-hydroxy-de-o-methyllasiodiplodin (**332**)	*Trichoderma* sp. 307	-	[64]
T39butenolide (**138**)	*T. harzianum* T22 *T. harzianum* T39	Antifungal	[23]	(3*R*,5*R*)-5-hydroxy-de-o-methyllasiodiplodin (**333**)	*Trichoderma* sp. 307	-	[64]
(22*E*,24*R*)-5α,8β-epidioxyergosta-6,22-dien-3-β-ol (**139**)	*T. harzianum* dl-36	-	[24]	(3*R*,6*R*)-6-hydroxy-de-o-methyllasiodiplodin (**334**)	*Trichoderma* sp. 307	-	[64]
ergosta-7,22-dien-3β,5α,6β-triol (**140**)	*T. harzianum* dl-36	-	[24]	(3*R*)-lasiodiplodin (**335**)	*Trichoderma* sp. 307	-	[64]
harzianic acid (**141**)	*T. harzianum* *Trichoderma* sp	Antibiotic Growth enhancing	[27] [65]	(3*S*)-ozoroalide (**336**)	*Trichoderma* sp. 307	-	[64]
15-hydroxyacorenone (**142**)	*T. harzianum*	-	[28]	(3*S*,5*R*)-5-hydroxylasiodiplodin (**337**)	*Trichoderma* sp. 307	-	[64]
2460A (**143**)	*T. harzianum*	Anti-tumor	[29]	(*E*)-9-etheno-lasiodiplodin (**338**)	*Trichoderma* sp. 307	-	[64]
trichokindin I (**144**)	*T. harzianum*	Bioinducer	[30]	(3*R*)-nordinone (**339**)	*Trichoderma* sp. 307	-	[64]
trichokindin II (**145**)	*T. harzianum*	Bioinducer	[30]	2,5-cyclohexadiene-1,4-dione-2,6-bis(1,1-dimethylethyl) (**340**)	*Trichoderma* sp. T-33	Antifungal	[68]
trichokindin III (**146**)	*T. harzianum*	Bioinducer	[30]	(*E*)-3-acetylbenzylbut-2-enoate (**341**)	*Trichoderma* sp. KK19L1	-	[69]
trichokindin IV (**147**)	*T. harzianum*	Bioinducer	[30]	1-hydroxy-6-methyl-9,10-anthraquinone (**342**)	*Trichoderma* sp. KK19L1	-	[69]
trichokindin V (**148**)	*T. harzianum*	Bioinducer	[30]	methyl hexadecanoate (**343**)	*Trichoderma* sp. 09	-	[70]
trichokindin VI (**149**)	*T. harzianum*	Bioinducer	[30]	N-2′-hydroxy-3′*E*-octadecenoyl-1-o-β-D-glucopyranosyl-9-methyl-4*E*,8*E*-sphingadiene (**344**)	*Trichoderma* sp. 09	Antifungal	[70]
trichokindin VII (**150**)	*T. harzianum*	Bioinducer	[30]	(4*E*,8*E*)-1-o-(β-D-glucopyranosyl)-2-(2′-hydroxyl-(*E*)-3′-heptadecenoylamideo)-3-hydroxyl-9-methyl-4,8-nonadecadiene (**345**)	*Trichoderma* sp. 09	Antifungal	[70]
trichorozin I (**151**)	*T. harzianum*	-	[31]	ergosta-7,24(28)-diene-3β-ol (**346**)	*Trichoderma* sp. 09	-	[70]
trichorozin II (**152**)	*T. harzianum*	-	[31]	cholest-4-ene-3-ol (**347**)	*Trichoderma* sp. 09	-	[70]
trichorozin III (**153**)	*T. harzianum*	-	[31]	methyl decanoate (**348**)	*Trichoderma* sp. 09	-	[70]
trichorozin IV (**154**)	*T. harzianum*	-	[31]	trichodin A (**349**)	*Trichoderma* sp. MF106	Antibiotic	[71]
octaketide keto diol (**155**)	*T. harzianum*	-	[32]	trichodin B (**350**)	*Trichoderma* sp. MF106	-	[71]
oxidized analog (**156**)	*T. harzianum*	-	[1]	pyridoxatin (**351**)	*Trichoderma* sp. MF106	Antibiotic	[71]
2-phenylethanol (**157**)	*T. harzianu*	-	[33]	trichodermone B (**352**)	*Trichoderma* sp. YLF-3	-	[73]
tyrosol (**158**)	*T. harzianum* *T. spirale* A17	Anti-tumor Hyperplasia-inhibitory	[33] [56]	3-(3-oxocyclopent-1-enyl)propanoic acid (**353**)	*Trichoderma* sp. YLF-3	Antibacterial	[73]
6-n-pentyl-•-pyrone (**159**)	*T. harzianum*	Antifungal Antibacterial	[33]	demethylsorbicillin (**354**)	*Trichoderma* sp. USF-2690	-	[74]
cyclo-(R-Pro-Gly) (**160**)	*T. harzianum*	-	[34]	oxosorbicillinol (**355**)	*Trichoderma* sp. USF-2690	DPPH-radical-scavenging	[74]
cyclo-(R-Pro-R-Ala) (**161**)	*T. harzianum*		[34]	alternariol 1′-hydroxy-9-methyl ether (**356**)	*Trichoderma* sp. Jing-8	Growth inhibitoring Antibacterial DPPH-radical-scavenging	[75]
cyclo-(S-Pro-R-Va1) (**162**)	*T. harzianum*	-	[34]	alternariol 9-methyl ether (**357**)	*Trichoderma* sp. Jing-8	-	[75]
cyclo-(4-methyl-R-Pro-S-Nva) (**163**)	*T. harzianum*	-	[34]	alternariol (**358**)	*Trichoderma* sp. Jing-8	DPPH-radical-scavenging	[75]
cyclo-(R-Pro-R-Leu) (**164**)	*T. harzianum*	-	[34]	altechromone A (**359**)	*Trichoderma* sp. Jing-8	-	[75]
cyclo-(R-Pro-R-Phe) (**165**)	*T. harzianum*	-	[34]	altenuene (**360**)	*Trichoderma* sp. Jing-8	-	[75]
cyclo-(4-hydroxyl-S-Pro-S-Leu) (**166**)	*T. harzianum*	-	[34]	4′-epialtenuene (**361**)	*Trichoderma* sp. Jing-8	-	[75]
uraci (**167**)	*T. harzianum*	-	[34]	scytalone (**362**)	*Trichoderma* sp. Jing-8	-	[75]
p-hydroxylphenylethanol (**168**)	*T. harzianum*	-	[34]	α-acetylorcinol (**363**)	*Trichoderma* sp. Jing-8	Growth inhibitoring	[75]
m-hydroxylphenylacitic acid (**169**)	*T. harzianum*	-	[34]	cerebroside C (**364**)	*Trichoderma* sp. Jing-8	-	[75]
3-dimethylamino-5-hydroxy-5-vinyl-2-cyclopenten-l-one (**170**)	*T. koningii*	-	[36]	α-palmitoyl-β-linoleoyl-α′-linoleoyl glycerol (**365**)	*Trichoderma* sp. Jing-8	-	[75]
7-*O*-methylkoninginin D (**171**)	*T. koningii*	-	[36]	1,2-benzenedicarboxylic acid bis(2*S*-methyl heptyl) ester (**366**)	*Trichoderma* sp. Jing-8	-	[75]
Trichodermaketone A (**172**)	*T. koningii*	Antifungal	[36] [37]	pachybasin (**367**)	*Trichoderma* sp. TA26-28	-	[76]
Trichodermaketone B (**173**)	*T. koningii*	-	[36]	chrysophanol (**368**)	*Trichoderma* sp. TA26-28	-	[76]
Trichodermaketone C (**174**)	*T. koningii*	-	[36]	8-*o*-methylchrysophanol (**369**)	*Trichoderma* sp. TA26-28	-	[76]
Trichodermaketone D (**175**)	*T. koningii*	-	[36]	soya-cerebroside I (**370**)	*Trichoderma* sp. TA26-28	-	[76]
koninginin A (**176**)	*T. koningii* *T. koningiopsis* Y10-2	-	[36] [43]	5α,6α-epoxyergosta-8(14),22-diene-3β,7α-diol (**371**)	*Trichoderma* sp. YM311505	-	[77]
koninginin D (**177**)	*T. koningii* *T. koningiopsis* Y10-2	-	[36] [43]	campesterol (**372**)	*Trichoderma* sp. YM311505	-	[77]
koninginin E (**178**)	*T. koningii*	-	[36]	7-methoxy-4,6-dimethyl phthalide (**373**)	*Trichoderma* sp. YM311505	Antibacterial Antifungal	[77]
koninginin F (**179**)	*T. koningii*	-	[36]	7-hydroxy-4,6-dimethyl phtalide (**374**)	*Trichoderma* sp. YM311505	-	[77]
6-pentyl-α-pyranone (**180**)	*T. koningii* T-8 *T. koningii* T-11	Antifungal	[38]	daidzein (**375**)	*Trichoderma* sp. YM311505	Antibacterial	[77]
6-(4-oxopentyl)-2*H*-pyran-2-one (**181**)	*T. koningii* T-8	Antifungal	[38]	cinnamic acid (**376**)	*Trichoderma* sp. YM311505	-	[77]
tricho-acorenol (**182**)	*T. koningii*	-	[39]	trichoacorenol (**377**)	*Trichoderma* sp. Xy24	-	[78]
methyl benzoate (**183**)	*T. koningii*	-	[40] [41]	trichocage B (**378**)	*Trichoderma* sp. Xy24	-	[79]
cyclo-(L-Pro-L-Leu) (**184**)	*T. koningii*	-	[40] [41]	1α-isopropyl-4α,8-dimethylspirod[4.5]-dec8-ene-2β,7α-di-ol (**379**)	*Trichoderma* sp. Xy24	-	[79]
4-hydroxyphenethylalcohol (**185**)	*T. koningii*	-	[40] [41]	1α-isopropyl-4α,8-dimethyl-spiro[4.5]dec-8-ene-3β,7α-diol (**380**)	*Trichoderma* sp. Xy24	-	[79]
ceramide (**186**)	*T. koningii*	-	[40] [41]	10,11-dihydroxy-cyclonerodiol (**381**)	*Trichoderma* sp. Xy24	-	[79]
trichokonin-V (**187**)	*T. koningii*	-	[40] [41]	14-hydroxy-trichoacorenol (**382**)	*Trichoderma* sp. Xy24	-	[79]
trichokonin-VI (**188**)	*T. koningii*	-	[40] [41]	harzianone (**383**)	*Trichoderma* sp. Xy24	-	[79]
trichokonin-II (**189**)	*T. koningii*	-	[40] [41]	(9*R*,10*R*)-dihydro-harzianone (**384**)	*Trichoderma* sp. Xy24	Cytotoxic	[79]
trichokonin-III (**190**)	*T. koningii*	-	[40] [41]	ergokonin B (**385**)	*Trichoderma* sp. Xy24	-	[79]
trichokonin-Ia (**191**)	*T. koningii*	-	[40] [41]	methyl stearate (**386**)	*Trichoderma* sp. Xy24	-	[79]
trichokonin-Ib (**192**)	*T. koningii*	-	[40] [41]	harzianelactone (**387**)	*Trichoderma* sp. Xy24	-	[80]
trichokonin-IX (**193**)	*T. koningii*	-	[40] [41]	trichoacorenol B (**388**)	*Trichoderma* sp. Xy24	-	[80]
trikoningin KAV (**194**)	*T. koningiopsis*	-	[1]	trichoacorenol C (**389**)	*Trichoderma* sp. Xy24	-	[80]
11-residue lipopeptaibols (**195**)	*T. koningiopsis*	-	[1]	cyclonerodiol B (**390**)	*Trichoderma* sp. Xy24	Anti-inflammatory	[80]
